# Individual, Social, and Environmental Correlates of Active Transportation Patterns in French Women

**DOI:** 10.1155/2017/9069730

**Published:** 2017-06-22

**Authors:** Camille Perchoux, Christophe Enaux, Jean-Michel Oppert, Mehdi Menai, Hélène Charreire, Paul Salze, Christiane Weber, Serge Hercberg, Thierry Feuillet, Franck Hess, Célina Roda, Chantal Simon, Julie-Anne Nazare

**Affiliations:** ^1^CRNH-Rhône-Alpes, CARMEN, INSERM U1060, Université de Lyon 1, INRA U1235, Lyon, France; ^2^Luxembourg Institute of Socio-Economic Research (LISER), Esch-sur-Alzette, Luxembourg; ^3^Laboratoire, Image, Ville et Environnement, Université de Strasbourg, Strasbourg, France; ^4^Equipe de Recherche en Epidémiologie Nutritionnelle (EREN), Centre de Recherche en Epidémiologie et Biostatistiques, INSERM U1153, INRA U1125, Cnam, COMUE Sorbonne Paris Cité, Université Paris 13, Bobigny, France; ^5^Department of Nutrition, Pitié-Salpêtrière Hospital (AP-HP), Institute of Cardiometabolism and Nutrition (ICAN), Université Pierre et Marie Curie-Paris 6, Paris, France; ^6^Department of Geography, Lab-Urba, Université Paris-Est, Créteil, France; ^7^Department of Public Health, Hôpital Avicenne (AP-HP), Bobigny, France; ^8^Department of Geography, UMR 7533, LADYSS, Université Paris 8, Saint-Denis, France

## Abstract

The objectives were (1) to define physical activity (PA) and sedentary behaviors (SB) patterns in daily life contexts (work, leisure, and transportation) in French working women from NutriNet-Santé web-cohort and (2) to identify pattern(s) of active transportation and their individual, social, and environmental correlates. 23,432 participants completed two questionnaires to evaluate PA and SB in daily life contexts and individual representations of residential neighborhood and transportation modes. Hierarchical cluster analysis was performed which identified 6 distinct movement behavior patterns: (i) active occupation, high sedentary leisure, (ii) sedentary occupation, low leisure, (iii) sedentary transportation, (iv) sedentary occupation and leisure, (v) active transportation, and (vi) active leisure. Multinomial logistic regressions were performed to identify correlates of the “active transportation” cluster. The perceived environmental characteristics positively associated with “active transportation” included “high availability of destinations around home,” “presence of bicycle paths,” and “low traffic.” A “positive image of walking/cycling,” the “individual feeling of being physically active,” and a “high use of active transport modes by relatives/friends” were positively related to “active transportation,” identified as a unique pattern regarding individual and environmental correlates. Identification of PA and SB context-specific patterns will help to understand movement behaviors' complexity and to design interventions to promote active transportation in specific subgroups.

## 1. Introduction

Physical inactivity is recognized as one of the main modifiable lifestyle risk factors for noncommunicable diseases such as cardiovascular disease, diabetes, and certain cancers [1, 2]. According to a recent worldwide report, about 23% of the European population does not meet WHO targets for sufficient physical activity (PA) for health [3]. Moreover, high levels of sedentary behavior such as prolonged sitting time have been associated with a deleterious health profile and mortality [4]. As demonstrated in a meta-analysis including more than 1 million individuals, jointly examining PA and sedentary behavior (SB) is of the upmost importance as high levels of PA may attenuate the detrimental health impact of prolonged sitting time [5]. This also demonstrates the ongoing need for an integrated approach to movement-related behaviors including both PA and SB.

Promoting active transportation (walking or cycling) appears as a relevant lever to increase usual PA at population level for several reasons: (1) active transportation has been associated with a higher level of daily total PA in cross-sectional and longitudinal studies [6–8]; (2) active transportation is an affordable, convenient, simple, and nonpolluting activity that can easily be integrated into everyday-life routines. However, designing effective PA interventions requires prior knowledge of the correlates (individual, environmental, and social) of usual PA [9].

Based on socioecological models of health behaviors, a large body of research has investigated the individual and environmental factors related to active transportation including individual-level factors such as age, body mass index (BMI), education level, self-efficacy, perceived benefits and barriers, employment status, and environmental-level factors such as street connectivity, walkability and cyclability, density of destination, traffic, population density, and social support [10–15]. Pioneering work in the early 2000s has put forward the need for context-specific analyses of the correlates of PA, more particularly for active transport.

During the course of a 24-hour day, movement-related behaviors alternate over time and PA (low-intensity, moderate to vigorous) and SB “compete” with each other depending on the contexts in which they are undertaken [10]. Therefore, analysis of the context-specific components of movement-related behavior, such as active transport, should not be performed in isolation but within the complex patterning of the various components of movement-related behaviors [5]. In this perspective, cluster analysis is gaining growing interest in health promotion research by providing valuable information on the way context-specific behaviors are corelated and organized relatively to each other in order to group individuals that present similar lifestyle patterns. Yet only few studies have investigated the patterns of movement-related behaviors in everyday-life contexts, using cluster or latent class analysis [16–20]. Such approaches are needed to identify the levers to be favored to promote active travel and developing more efficient tailored prevention programs.

Our objectives were (1) to define PA and SBs patterns in daily life contexts (work, leisure, and transportation) in a large sample of French adult women from an ongoing web-cohort, (2) to identify pattern(s) of active transportation, and to (3) to investigate, through a socioecological approach, the individual, social, and environmental correlates of active transportation within comprehensive movement-related behaviors patterns.

## 2. Subjects and Methods

### 2.1. Population

This study is part of the ACTI-Cités project [21] based on the Nutrinet-Santé Cohort Study, a web-based cohort launched in France in 2009 to evaluate the relationships between nutrition and health [22]. In the ACTI-Cités study, participants of the Nutrinet-Santé Cohort Study aged 18 years or older were invited to complete two web-based questionnaires, as previously reported [10, 23]. The Sedentary and Transport Activity Questionnaire (STAQ) [24] assessed the last 4-week PA and SBs in daily life contexts including work, leisure, and transportation. The questionnaire of the daily life environment (QEVIC) [25] evaluated the individual's representations of their residential neighborhood and transportation modes. This study was conducted according to the guidelines laid down in the Declaration of Helsinki, and all procedures were approved by the Institutional Review Board of the French Institute for Health and Medical Research (IRB Inserm number 0000388FWA00005831) and the* Commission Nationale Informatique et Libertés* (CNIL number 908450 and no 909216). All participants gave their written electronic informed consent to take part in the study. The present analysis is based on the women subsample of the ACTI-Cités study (71.3% women).

### 2.2. Variables of Physical Activity and Sedentary Behaviors

Five variables from the STAQ were computed to evaluate the PA and SB in everyday-life contexts [24].

#### 2.2.1. Physical Activity

Participants were asked to report the past-month frequency, duration, and modes of transportation in different contexts including commuting and utilitarian purposes. The time spent active during transportation (hours/week) was calculated by summing the time per week spent walking, cycling, or using another active transportation mode (i.e., skateboard, etc.). Participants were also asked to report the past-month frequency and duration of 38 types of sport and other active leisure activities. The active time spent during leisure (hours/week) was calculated by summing the time spent per week doing these 38 activities. Participants were also asked to evaluate whether they perceived themselves as physically active individuals (yes/no).

#### 2.2.2. Sedentary Behaviors

Participants were asked to report the average time per workday and nonworkday they spent watching TV, using a computer or a tablet computer or playing videogames, reading, writing, knitting, or other sedentary leisure activities, during the past month. The sedentary time spent during leisure (hours/week) was calculated by summing the time per week spent in each reported sedentary leisure activity. Similarly to the computation of active transportation time, the time spent sedentary during transportation (hours/week) was calculated by summing the time spent in an individual motorized transportation mode (i.e., car, motorbike) and/or using public transports. The time spent sedentary at work (hours/week) was assessed by the mean length of time per workday spent sitting at work, during the past month.

### 2.3. Environmental, Social and Individual Variables

#### 2.3.1. Perceived Residential Environment Attributes

Information regarding the perceived residential environment was obtained using the QEVIC [25]. We considered the following variables: availability of target destinations, ease of walking on sidewalks, presence of bicycle paths, pollution levels of the neighborhood, cleanliness/maintenance of the neighborhood, presence of trees, presence of greenspace, neighborhood aesthetics, traffic, and criminality.

#### 2.3.2. Individual Representation of Transportation Modes

The individual's representations of each transportation mode including walking, cycling, individual motorized transportation modes, and public transport were assessed from the QEVIC [25].

#### 2.3.3. Use of Active Transportation Modes by Relatives and Friends

Family's and friends' use of active transportation modes was assessed from the QEVIC [25].

#### 2.3.4. Physical Activity Value by the Family

Participants were also asked in the STAQ whether their family valued PA and more specifically sports.

Detailed information on items of the QEVIC and STAQ questionnaires, variables' computation, and Cronbach's alpha are provided in Supplementary Table 1 in Supplementary Material available online at https://doi.org/10.1155/2017/9069730.

### 2.4. Covariates

Several sociodemographic variables were considered for adjustment: age, individual education level, having a child under the age of 13 within the household, and living in an urban or a rural setting. Age was used as a continuous variable. Individual education was divided into four classes (no education or primary education, secondary education, lower tertiary education, and upper tertiary education).

### 2.5. Statistical Analyses

First, we conducted a hierarchical cluster analysis using Ward's method to define a typology of patterns of movement. This method has been used previously in analyses on habitual PA (see [16, 17]). The cluster analysis accounted for 5 variables of PA and SB in context, including time spent sedentary at work (hours/week), time spent sedentary during leisure activities (hours/week), time spent active during leisure (hours/week), time spent sedentary during transportation (hours/week), and time spent active during transportation (hours/week). We tested solutions between 4 and 8 clusters. Based on the dendrogram and the distribution of the subjects between clusters, we chose a 6-cluster solution representing contrasted patterns of movement with over half of the variation in the five SBs and PA variables being accounted for (*R*^2^ = 0.51).

Second, we used multinomial logistic regression analysis to identify the correlates of the 6 patterns of movement, using the cluster with the highest level of active transportation as reference (cluster 5). Odds ratios (OR) and 99% confidence interval (CI) were computed. All variables related to the representation of residential environment, transportation modes, use of active transportation modes by friends and family, and representation of PA were tested for multicollinearity using variance inflation factor [26]. No multicollinearity between variables was detected (all VIF < 2). All variables were first tested one-by-one in unadjusted models. We then combined into one model the variables that were independently associated with at least one cluster controlled for age, individual education, having a child under the age of 13 in the household, and living in an urban versus a rural setting. Finally, we only retained variables that were associated with at least one cluster in the multivariate model.

Sensitivity analysis was performed to evaluate the stability of the cluster analysis. Hierarchical cluster analysis was replicated on a random 10% of the initial sample to evaluate whether participants similarly aggregated in subsamples [17]. All analyses were performed with SAS 9.4 (SAS Inst., Cary, NC, USA).

## 3. Results

### 3.1. Characteristics of the Study Population

Among 38,913 women who filled in the STAQ and the QEVIC in 2013, 619 women were excluded for self-reported motor impairments (*N* = 619) and/or for self-reported limitations on walking 100 m (*N* = 562) and/or for reporting implausible PA or sedentary activity values (i.e., extreme reported values above the 99.5 percentile for each PA and SB activity) (*N* = 1630). Additionally, we excluded 12,864 participants who did not report a work or study activity, or who were retired. The final sample included 23,432 participants. Descriptive information on the sample (demographics, context-specific PA and SB) is provided in Table 1.

### 3.2. Clusters of Physical Activity and Sedentary Behaviors by Daily Life Contexts

Descriptive statistics for the six identified clusters including both active and sedentary behaviors are presented in Table 2.* Cluster 1* (30.3% of the population) was mainly characterized by the lowest duration of SB at work. Individuals in this cluster also accumulated high relative levels of leisure time. This cluster was labeled “*active occupation, high sedentary leisure*.”* Cluster 2* (50.8% of the population) mainly captured highly sedentary individuals at work. Individuals in this cluster also accumulated low absolute and relative levels of leisure time. This cluster was labeled “*sedentary occupation, low leisure*.”* Cluster 3* (3.3% of the population) had the highest duration of sedentary transportation and the second highest duration for sedentary leisure time activities. This cluster was labeled “*sedentary transportation*.”* Cluster 4* (8.0% of the population) had the highest duration of sedentary leisure activities and the second highest duration of SB at work. This cluster was labeled “*sedentary occupation and leisure*.”* Cluster 5* (5.5% of the population) had the highest duration of active transportation and the second highest duration in active leisure. This cluster was labeled “*active transportation*.”* Cluster 6* (2.1% of the population) had the highest duration in active leisure. Participants in this cluster also accumulated the second highest duration in sedentary leisure. This cluster was labeled “*active leisure*.”

Sensitivity analysis based on a random sample representing 10% of the initial sample showed similar patterns of movement as the whole sample, with a relatively strong agreement (Cramer's *V* = 0.69). Characteristics of study participants by clusters are presented in Table 3.

### 3.3. Association between Patterns of Movement Behaviors and Perceived Environmental Characteristics, Representation of Transportation Modes, and Physical Activity

Cluster 5 “*active transportation*” was used as reference since it represented the highest level of active transport, as well as the highest percentage of individuals who reported active transportation. Unadjusted associations between clusters and perceived environmental characteristics, representation of transportation modes, friends and relatives' use of active transportation modes, and PA (not adjusted for each other) are provided in Supplementary Table 2. Multivariate associations are presented in Table 4.

#### 3.3.1. Cluster 1 “Active Occupation, High Sedentary Leisure” versus Cluster 5 “Active Transportation”

Among the perceived environmental characteristics, a high availability of destinations and a high presence of bicycle paths strongly decreased the odds of belonging to Cluster 1 “*active occupation, high sedentary leisure*” compared with the referent Cluster 5. A perceived highly polluted and low aesthetic residential neighborhood decreased the odds of belonging to Cluster 1. A high positive representation of walking and biking and inversely a low positive representation of individual motorized transportation modes and representing oneself as an active person decreased the odds of belonging to Cluster 1. As for social environment, the likelihood of belonging to Cluster 1 was negatively associated with a high use of walking and cycling among relatives.

#### 3.3.2. Cluster 2 “Sedentary Occupation, Low Leisure” versus Cluster 5 “Active Transportation”

Cluster 2* “sedentary occupation, low leisure”* presented the same associations with the perception of residential environment and social environment, as Cluster 1 compared with Cluster 5. Moreover, as for this latter, representing oneself as an active person decreased by 50% the odds of belonging to Cluster 2.

#### 3.3.3. Cluster 3 “Sedentary Transportation” versus Cluster 5 “Active Transportation”

Perceiving a high availability of destinations and bicycle paths, having a high positive representation of walking, cycling, and using public transports, having relatives using active transportation modes, and representing oneself as an active person were associated with a decrease in the odds of being in Cluster 3* “sedentary transportation”* compared with Cluster 5.

#### 3.3.4. Cluster 4 “Sedentary Occupation and Leisure” versus Cluster 5 “Active Transportation”

No perceived residential environment variables were correlated to the probability of belonging to Cluster 4 compared with Cluster 5. Cluster 4 differed from Cluster 5 in representation of transportation modes, family's use of active transportation modes, and self-representation of being an active person.

#### 3.3.5. Cluster 6 “Active Leisure” versus Cluster 5 “Active Transportation”

A high presence of bicycle paths and having friends and family members regularly using active transportation modes were associated with a strong decrease in the odds of being in Cluster 6 compared with Cluster 5. In contrast, living in a perceived low pollution and highly aesthetic neighborhood and having a positive representation of individual motorized transportation modes were associated with an increase in the odds of being in Cluster 6.

## 4. Discussion

In this study of a large sample of French working women, cluster analyses of detailed context-specific PA and SB data revealed an original typology of 6 distinct movement behavior patterns in daily life contexts. A specific cluster characterizing individuals with high levels of active transportation was identified as a unique pattern regarding individual and environmental correlates.

The present analysis highlights the variety of existing combinations of PA and SB according to contexts such as work, transportation, or leisure. Context-specific physical or sedentary activities have been related to distinct health effects [16, 27, 28] and their cooccurrence could have a synergistic or antagonistic impact on health risk profile. This reinforces the importance of comprehensive analyses of daily life activities patterns and their correlates, to identify at-risk behavioral profiles for chronic diseases and mortality [5, 29].

As previously reported [17, 18, 20], we observed that activities are not exclusive nor simply in opposition but rather cooccur throughout the day. For instance, the* “sedentary transportation”* cluster presents the second highest time spent in active transportation. This mixed pattern has also been observed in previous works [30, 31], as those who report high overall transportation time may have more opportunity of mixing passive and active means. The* “active leisure”* cluster and* “active transportation”* cluster presented the highest levels of reported PA (excluding occupational), consistent with previous reports from both US and French cohorts [17, 18]. Cross-sectional and longitudinal studies that investigated the association between leisure and transportation physical activities suggested that higher levels of active transportation are related to higher level of overall and leisure PA and not to a substitution of one context-related activity by another [6, 14, 32]. In our study, clusters with the lowest active transportation time also displayed the lowest time spent active in total or during leisure, but not necessarily the highest time spent sedentary. This confirms that interventions to promote active transportation should take into account the overall movement-related behavior of individuals, targeting both sedentary and active components accordingly.

In this sense, it is well recognized that context-specific PA [9, 33, 34] and SB [35, 36] relate to distinct factors. Nevertheless, only few studies have simultaneously addressed the different components of movement behavior, by either focusing on one component and adjusting for another or by separately examining patterns of SB et PA [16, 17, 20, 29, 37]. Presently, in order to analyze active transportation behavior, we have specifically used cluster analysis to capture integrative patterns and to investigate the correlates of active transportation behavior within global movement activities rather than as an isolated behavior.

An important finding in this study is that the correlates of one of the clusters, termed “active transportation,” were clearly distinct from those of all the other 5 clusters. This was evidenced through a detailed investigation of individual, social, and environmental factors that could differentiate the “active transportation” cluster from the other patterns of movement. The perceived environmental characteristics positively associated with the probability of belonging to the cluster* “active transportation”* as opposed to other clusters include high availability of destinations around the home, the presence of bicycle paths, and low traffic. Unexpectedly, the perception of a polluted residential area and a perceived low aesthetic environment were also positively associated with the cluster* “active transportation.”* Such inverse associations have been observed elsewhere and may reflect that highly walk/bike-friendly environments are likely to be located in urban areas concentrating high multimodal transportation facilities and thus highly traffic polluted [38, 39]. As previously documented, active transportation has been associated with environment features [40], such as higher residential density, mixed land use, street connectivity, access to destinations, and walking/cycling facilities [40, 41], with mixed results regarding aesthetics [11, 12] or safety [40, 42–44]. Previous literature also suggests that physical environmental attributes may be more related to active transportation than active leisure [45]. Concomitantly, environmental factors were strong correlates of belonging to the “active leisure” cluster compared to the “active transportation” cluster. Our results confirm the crucial link between perceived environment and active transportation pattern.

Regarding social factors, a high use of active transportation modes by relatives and friends was significantly associated with all patterns of movements compared to the* “active transportation”* cluster, this being even stronger for a family's use. When reviewing the social determinants of active travel in adults, Panter and Jones reported that active commuting was associated with social support from family and friends in Europe, with less consistency in Australia or the US [31]. In Europe, relatives' level of PA, walking/cycling with siblings or friends, was positively associated with total active transportation as well as social norms, social modelling, and social support [46–48].

We also investigated the link between the representation of different transportation modes and PA on patterns of movement. A positive representation of walking/cycling, a negative representation of individual motorized transportation, and the individual feeling of being physically active were positively associated with the probability of belonging to the cluster* “active transportation.”* Longitudinal findings from the UK reported that alternatives to car use could be predicted by a lower favorable attitude towards the car [30].

In our study, compared to the referent cluster, the* “sedentary transportation”* cluster was not associated with representation of individual motorized transport modes but was negatively associated with a positive representation of public transport. Of note, this cluster displayed the highest time spent for transportation and second highest time for active transportation. This suggests that sedentary and active transportation behaviors are not exclusive or opposed and can cooccur more particularly in individuals reporting high transportation time.

Overall, the directions of the associations with the different studied factors were mainly similar for all clusters compared to the* “active transportation”* cluster, although we observed some slight variations in the significance and the strength of these associations from one cluster to another.

## 5. Strengths and Limitations

The originality of this study lies in the socioecological approach to the active transportation pattern's correlates as an integrated combination of daily life activities. One other strength is the large sample size and the specific validated questionnaires on the time spent in PA and SB in daily life contexts [24], with a detailed assessment of the perception of environment and transportation modes.

Nevertheless, our study presents some limitations. Its cross-sectional design prevents drawing any causal relationships. Self-reported questionnaires rather than objective measures were used to assess both context-specific PA and SB which could be a source of potential biases. It has been evidenced that sedentary SB can displace physical activity time, both moderate to vigorous (MVPA) and light intensity (LPA) [20, 29]. Physical activities encompass MVPA and LPA that also are interdependent in terms of time allocated and health effects [29, 37, 49]. Presently we did not subdivide LPA and MVPA. The workplace or leisure environments were not taken into account resulting in potential environment misrepresentation [50]. The analysis was performed on a female sample, included on a voluntary basis with relatively more highly educated individuals compared to the National Census data and with a large majority residing in urban areas [51], limiting extrapolation of the results to other populations. Preferences regarding residential location were not assessed, precluding residential neighborhood self-selection to be taken into account [52].

## 6. Conclusion

The identification of patterns of context-specific PA and SB is a step forward in the understanding of the complexity of movement behaviors towards a refined approach of active transportation and related correlates. Targeting pattern-specific correlates will help to design dedicated intervention programs for active transportation and physical activity in specific subgroups.

## Supplementary Material

Supplementary Table 1: Description of the socioecological variables. Supplementary Table 2: Unadjusted associations between perceived environmental characteristics, representation of transportation modes, physical activity, and clusters of physical activity and sedentary behaviors. Results from multinomial logistic regression model using cluster 5: active transportation as a reference (N = 23432).

## Figures and Tables

**Table 1:** Characteristics of the study population (*N* = 23,432 women).

Variables	% or mean	SD

Sociodemographics		
Age (mean, years)	42.6	(11.5)
Living with a partner (*%*)	69.9	—
Living with a child under the age of 13 (*%*)	30.9	—
Individual education level (*%*)		
* High*	38.8	—
* Middle-high *	34.0	—
* Middle-low *	17.9	—
* Low *	9.4	—
Professional status (*%*)		
* Farmer*	0.4	—
* Craftsperson, company head*	1.8	—
* Executive (liberal profession, engineer)*	32.1	—
* Intermediate profession (technician, teacher, and supervisor)*	28.1	—
* Employee*	30.0	—
* Laborer worker*	1.4	—
* Other*	6.2	—
Living in a urban setting (*%*)	89.7	—
Physical activity and sedentary behavior by context		
Leisure		
Leisure time spent active (h/week)	2.7	(3.9)
Leisure time spent sedentary (h/week)	33.8	(21.9)
Total leisure time (h/week)	36.5	(22.2)
Transportation		
Time spent walking/cycling for transportation (h/week)	2.2	(4.4)
Time spent sedentary for transportation (h/week)	2.0	(3.5)
Total transportation time (h/week)	4.2	(5.9)
Work		
Time spent sedentary at work (h/week)	21.9	(15.1)
Total active time (h/week)	5.1	(6.2)
Total sedentary time (h/week)	57.5	(28.4)

SD: standard deviation.

**Table 2:** Description of the clusters of physical activity and sedentary behaviors by daily life contexts (*N* = 23,432).

	Cluster 1		Cluster 2		Cluster 3		Cluster 4		Cluster 5		Cluster 6		Fisher's *F-*test or Chi^2^* p *value
	Active occupation, high sedentary leisure		Sedentary occupation, low leisure		Sedentary transportation		Sedentary occupation and leisure		Active transportation		Active leisure		
	*n* = 7088		*n* = 11897		*n* = 781		*n* = 1865		*n* = 1299		*n* = 502		
	(30.3%)		(50.8%)		(3.3%)		(8.0%)		(5.5%)		(2.1%)		
	Mean	(SD)	Mean	(SD)	Mean	(SD)	Mean	(SD)	Mean	(SD)	Mean	(SD)	
Leisure time spent active (hours/week)	2.5	(2.5)^*∗*^	2.4	(2.5)^*∗*^	3.9	(4.5)^*∗*^	1.5	(1.4)^*∗*^	4.5	(4.5)	19.8	(7.8)^*∗*^	<0.0001
Leisure time spent sedentary (hours/week)	28.4	(15.8)	29.7	(16.6)	36.3	(22.1)^*∗*^	79.3	(22.3)^*∗*^	30.1	(17.3)	34.2	(21.3)^*∗*^	<0.0001
Total leisure time (hours/week)	30.8	(15.8)^*∗*^	32.2	(17.1)^*∗*^	40.2	(22.4)^*∗*^	80.8	(22.3)^*∗*^	34.6	(18.2)	54.0	(22.6)^*∗*^	<0.0001
Time spent walking/cycling for transportation (hours/week)	1.0	(1.4)^*∗*^	1.5	(1.9)^*∗*^	4.0	(7.6)^*∗*^	2.2	(2.8)^*∗*^	14.9	(9.0)	1.8	(2.5)^*∗*^	<0.0001
Time spent sedentary for transportation (hours/week)	1.7	(1.9)^*∗*^	1.4	(1.8)^*∗*^	16.7	(6.4)^*∗*^	1.2	(1.4)^*∗*^	2.0	(2.3)	1.6	(2.0)^*∗*^	<0.0001
Total transportation time^a^ (hours/week)	2.6	(2.3)^*∗*^	2.9	(2.6)^*∗*^	20.7	(10.5)^*∗*^	3.4	(3.2)^*∗*^	17.0	(9.4)	3.4	(3.2)^*∗*^	<0.0001
Time spent sedentary at work (hours/week)	5.7	(5.5)^*∗*^	30.7	(9.9)^*∗*^	18.7	(14.8)^*∗*^	34.9	(12.0)^*∗*^	16.4	(14.0)	14.6	(12.4)^*∗*^	<0.0001
Total time spent active^a^ (hours/week)	3.4	(2.9)^*∗*^	3.9	(3.2)^*∗*^	7.9	(9.4)^*∗*^	3.7	(3.3)^*∗*^	19.4	(10.5)	21.6	(8.5)^*∗*^	<0.0001
Total time spent sedentary^a^ (hours/week)	35.7	(16.9)^*∗*^	61.9	(18.5)^*∗*^	71.6	(29.0)^*∗*^	115.4	(23.2)^*∗*^	48.5	(22.8)	50.4	(26.4)	<0.0001

^*∗*^Significant pairwise least-square means differences from “Cluster 5, active transportation,” with Tukey-Kramer adjustment at  *p* < 0.01. ^a^Total time spent during transportation, active and sedentary, was not included within cluster analysis. SD: standard deviation.

**Table 3:** Characteristics of study participants by clusters of physical activity and sedentary behaviors (*N* = 23,432).

	Cluster 1	Cluster 2	Cluster 3	Cluster 4	Cluster 5	Cluster 6	Chi^2^ *p *value
	Active occupation, high sedentary leisure	Sedentary occupation, low leisure	Sedentary transportation	Sedentary occupation and leisure	Active transportation	Active leisure	
	*n* = 7088	*n* = 11897	*n* = 781	*n* = 1865	*n* = 1299	*n* = 502	
	(30.3%)	(50.8%)	(3.3%)	(8.0%)	(5.5%)	(2.1%)	
Age^a^*(mean (SD))*	43.8 (11.7)^*∗*^	41.7 (11.2)^*∗*^	43.8 (11.2)^*∗*^	39.6 (11.6)^*∗*^	45.6 (12.2)	48.0 (11.2)^*∗*^	<0.0001^†^
Living with a partner (*%*)	73.5	69.9	72.2	57.9	64.8	71.3	<0.0001
Having a child under the age of 13 (*%*)	31.7^*∗*^	32.3^*∗*^	38.4^*∗*^	23.4	22.7	24.7	<0.0001
Individual education level^b^ (*%*)							<0.0001
* High*	27.5	45.8	29.9	46.5	32.0	31.1	
* Middle high*	38.1	32.5	35.4	28.1	33.1	35.1	
* Middle-low*	20.5	15.5	22.8	18.6	20.7	19.5	
* Low*	13.9	6.2	12.0	6.8	14.3	14.3	
Professional status (%)							<0.0001
* Farmer*	0.7	0.1	0.4	0.0	0.9	1.2	
* Craftsperson, company head*	2.5	1.3	2.2	1.8	2.1	2.6	
* Executive (liberal profession, engineer)*	20.7	39.1	25.4	36.3	28.1	31.3	
* Intermediate profession (technician, teacher, and supervisor)*	35.2	25.3	31.0	19.4	25.6	29.7	
* Employee*	33.1	27.1	34.7	31.5	35.3	29.5	
* Laborer*	2.6	0.7	1.5	1.3	1.3	1.8	
Urban setting (versus rural) (*%*)	86.8^*∗*^	90.6^*∗*^	85.9^*∗*^	93.7	94.8	84.7^*∗*^	<0.0001

^*∗*^Estimate significantly different from “Cluster 5, active transportation,” at *p* < 0.01, using Fisher's *F*-test. ^†^Fisher's *F*-test *p* value. ^a^Missing values (*n* = 3). ^b^Missing values (*n* = 182). SD: standard deviation.

**Table 4:** Adjusted associations between perceived environmental, representation of transportation modes, physical activity, and clusters of physical activity and sedentary behaviors. Results from multinomial logistic regression model using Cluster 5, active transportation as reference (*N* = 23,222^*∗*^).

	Cluster 1		Cluster 2		Cluster 3		Cluster 4		Cluster 6	
	Active occupation, high sedentary leisure		Sedentary occupation, low leisure		Sedentary transportation		Sedentary occupation and leisure		Active leisure	
	OR	(CI 99%)	OR	(CI 99%)	OR	(CI 99%)	OR	(CI 99%)	OR	(CI 99%)
Perception of the residential environment *(high *versus* low)*										
Availability of destinations	**0.64**	**(0.54; 0.77)**	**0.76**	**(0.64; 0.90)**	**0.64**	**(0.49; 0.82)**	0.94	(0.76; 1.16)	0.84	(0.63; 1.12)
Presence of bicycle paths	**0.66**	**(0.56; 0.78)**	**0.75**	**(0.64; 0.89)**	**0.70**	**(0.53; 0.91)**	0.81	(0.66; 1.00)	**0.69**	**(0.51; 0.93)**
Perceived pollution	**0.74**	**(0.62; 0.88)**	**0.82**	**(0.69; 0.97)**	0.77	(0.59; 1.00)	0.95	(0.76; 1.16)	**0.68**	**(0.50; 0.94)**
Aesthetics	**1.30**	**(1.08; 1.57)**	**1.37**	**(1.08; 1.57)**	1.33	(1.00; 1.78)	1.18	(0.95; 1.47)	**1.31**	**(0.93; 1.84)**
Positive representation of transportation modes *(high *versus *low)*										
Walking	**0.56**	**(0.46; 0.68)**	**0.58**	**(0.48; 0.69)**	**0.50**	**(0.36; 0.69)**	**0.63**	**(0.49; 0.79)**	0.91	(0.65; 1.26)
Cycling	**0.83**	**(0.69; 0.99) **	**0.73**	**(0.61; 0.87)**	**0.73**	**(0.56; 0.95)**	**0.64**	**(0.52; 0.80)**	0.91	(0.67; 1.24)
Individual motorized transportation modes	**1.40**	**(1.18; 1.67)**	**1.23**	**(1.04; 1.45)**	1.26	(0.98; 1.62)	**1.35**	**(1.10; 1.66)**	**1.55**	**(1.17; 2.06)**
Public transport	0.91	(0.77; 1.08)	1.03	(0.87; 1.22)	**0.77**	**(0.60; 0.98)**	0.99	(0.80; 1.21)	0.83	(0.62; 1.11)
Friends and relatives' use of active transportation modes *(high *versus *low)*										
Family's use of walking/cycling for transportation	**0.54**	**(0.43; 0.66)**	**0.53**	**(0.43; 0.65)**	**0.51**	**(0.37; 0.68)**	**0.56**	**(0.44; 0.72)**	**0.51**	**(0.36; 0.73)**
Friends' use of walking/cycling for transportation	**0.79**	**(0.64; 0.96)**	**0.86**	**(0.64; 0.96)**	**0.71**	**(0.53; 0.96)**	0.83	(0.65; 1.06)	**0.77**	**(0.55; 1.09)**
Representation of physical activity										
Self-representation of being active (*yes *versus *no*)	**0.74**	**(0.62; 0.88)**	**0.50**	**(0.42; 0.59)**	**0.64**	**(0.50; 0.82)**	**0.32**	**(0.26; 0.39)**	1.23	(0.90; 1.69)

*Note*. Model adjusted on urban versus rural setting, age, education, and having a child under the age of 13. The following variables were not statistically significant in the adjusted multivariate model: perceived walkability, perception of green spaces, presence of trees, perceived traffic, perceived criminality, perceived cleanliness of the neighborhood, and family representation of physical activity. ^*∗*^185 missing values related to education (*n* = 182) and age (*n* = 3). CI: confidence interval; OR: odds ratio.
