# Supplementary material for: Individual, Social, and Environmental Correlates of Active Transportation Patterns in French Women
Source: Biomed Res Int. 2017 Jun 22;2017:9069730. doi: 10.1155/2017/9069730 (PMC5498903; doi:10.1155/2017/9069730)
Supplement: Supplementary file 1 — Supplementary Table 1: Description of the socioecological variables. Supplementary Table 2: Unadjusted associations between perceived environmental characteristics, representation of transportation modes, physical activity, and clusters of physical activity and sedentary behaviors. Results from multinomial logistic regression model using cluster 5: active transportation as a reference (N = 23432). [file 9069730.f1.pdf]

Supplementary Table 1: Description of the socio-ecological variables

VARIABLES	QUESTIONS, ANSWERS AND ITEMS*	COMPUTATION OF THE VARIABLE	CATEGORICAL VARIABLES' THRESHOLDS
Perceived residential environment attributes			
Availability of destination *(high vs. low)*	Around your home you have access to: (multiple choice)<br>□ A small supermarket<br>□ A grocery store<br>□ A bank<br>□ A post office<br>□ A school<br>□ A bakery<br>□ A restaurant<br>□ A cafe terrace<br>□ A pharmacy<br>□ None of the above	Sum of destinations ranking from 0 to 9.	Binary variable categorized at the median value (threshold=7).
Ease to walk on sidewalks *(high vs. low)*	Among the following proposals, choose the one that best describes how you can walk around your home<br>a - You can walk on the sidewalks without being hindered by other pedestrians, cyclists or parked cars.<br>b - You can walk on the sidewalks, but you are sometimes hindered by other pedestrians, cyclists or parked cars.<br>c- On the sidewalks, you are often forced to walk around obstacles or to walk on the road.<br>d - There are no sidewalks around my home<br>e - You're not concerned	-	Binary variable categorized as high (a, b) versus low (c, d).<br>The "e" answer has not been evaluated.
Presence of bicycle paths *(high vs. low)*	Among the following proposals, choose the one that best describes how it is possible to move by bike around your home<br>a - There are bike lanes separated from the road<br>b - There are bike lanes on roads<br>c - You must take the road without bike lanes<br>d - You don't know	-	Binary variable categorized as high (a, b) versus low (c).<br>The "d" answer has not been evaluated.

Pollution<br>*(high vs. low)*	The neighborhood is polluted:<br>a - Strongly agree<br>b - Agree<br>c - Neither agree nor disagree<br>d - Disagree<br>e - Strongly disagree		Binary variable categorized as high (a, b) versus low (c, d, e).
Cleanliness of the neighborhood<br>*(high vs. low)*	The neighborhood is clean and well:<br>a - Strongly agree<br>b - Agree<br>c - Neither agree nor disagree<br>d - Disagree<br>e - Strongly disagree	-	Binary variable categorized as high (a, b) versus low (c, d, e).
Presence of trees<br>*(high vs. low)*	There are trees along the streets of the neighborhood:<br>a - Strongly agree<br>b - Agree<br>c - Neither agree nor disagree<br>d - Disagree<br>e - Strongly disagree	-	Binary variable categorized as high (a, b) versus low (c, d, e).
Presence of green spaces<br>*(high vs. low)*	There is one or more green spaces in the neighborhood:<br>a - Strongly agree<br>b - Agree<br>c - Neither agree nor disagree<br>d - Disagree<br>e - Strongly disagree	-	Binary variable categorized as high (a, b) versus low (c, d, e).
Aesthetics<br>*(high vs. low)*	There are a lot of poorly maintained, unoccupied or ugly buildings in the neighborhood:<br>a - Strongly agree<br>b - Agree<br>c - Neither agree nor disagree<br>d - Disagree<br>e - Strongly disagree	-	Binary variable categorized as high (d, e) versus low (a, b, c).
Traffic<br>*(high vs. low)*	Your home neighborhood is not safe to walk because of road traffic<br>a - Strongly agree<br>b - Agree		Binary variable categorized as high (a, b) versus low (c, d, e).

	c - Neither agree nor disagree<br>d - Disagree<br>e - Strongly disagree		
Criminality<br>*(high vs. low)*	<u>During the day, the neighborhood is not safe because of criminality</u><br>a - Strongly agree<br>b - Agree<br>c - Neither agree nor disagree<br>d - Disagree<br>e - Strongly disagree<br><br><u>At night, the neighborhood is not safe because of criminality</u><br>a - Strongly agree<br>b - Agree<br>c - Neither agree nor disagree<br>d - Disagree<br>e - Strongly disagree	Mean of the two item questions ranking from 1 (Strongly agree) to 5 (strongly disagree).<br>(Cronbach's α=0.82)	Binary variable categorized at the median value (threshold=0.2).
**Positive representations of transportation modes**			
Walking<br>*(high vs. low)*	<u>Walking is:</u><br>□ Fast Very/Pretty/Not<br>□ Comfortable Very/Pretty/Not<br>□ Cheap Very/Pretty/Not<br>□ Safe, Secure Very/Pretty/Not<br>□ Convenient Very/Pretty/Not<br>□ Relaxing Very/Pretty/Not	Each item was rated, ranking from 1 (very) to 3 (Not).<br>A score was computed by calculating the average of the 6 items.<br>(Cronbach's α=0.77)	Binary variable categorized at the median value (threshold=0.53).
Cycling<br>*(high vs. low)*	<u>Bike is:</u><br>□ Fast Very/Pretty/Not<br>□ Comfortable Very/Pretty/Not<br>□ Cheap Very/Pretty/Not<br>□ Safe, Secure Very/Pretty/Not<br>□ Convenient Very/Pretty/Not<br>□ Relaxing Very/Pretty/Not<br>□ Green Very/Pretty/Not	Each item was rated, ranking from 1 (very) to 3 (Not).<br>A score was computed by calculating the average of the 7 items.<br>(Cronbach's α=0.77)	Binary variable categorized at the median value (threshold=0.53).
Individual motorized	<u>Car is:</u>	Each item was rated, ranking	Binary variable categorized

transportation modes<br>*(high vs. low)*	□ Fast Very/Pretty/Not<br>□ Comfortable Very/Pretty/Not<br>□ Cheap Very/Pretty/Not<br>□ Safe, Secure Very/Pretty/Not<br>□ Convenient Very/Pretty/Not<br>□ Relaxing Very/Pretty/Not<br>□ Green Very/Pretty/Not<br><br><u>Motorcycle is:</u><br>□ Fast Very/Pretty/Not<br>□ Comfortable Very/Pretty/Not<br>□ Cheap Very/Pretty/Not<br>□ Safe, Secure Very/Pretty/Not<br>□ Convenient Very/Pretty/Not<br>□ Relaxing Very/Pretty/Not<br>□ Green Very/Pretty/Not	from 1 (very) to 3 (Not).<br>A score was computed by calculating the average of the 14 items.<br>(Cronbach's α=0.72)	at the median value (threshold=0.71).
Public transport<br>*(high vs. low)*	<u>Tram/suburban train/subway is:</u><br>□ Fast Very/Pretty/Not<br>□ Comfortable Very/Pretty/Not<br>□ Cheap Very/Pretty/Not<br>□ Safe, Secure Very/Pretty/Not<br>□ Convenient Very/Pretty/Not<br>□ Relaxing Very/Pretty/Not<br>□ Green Very/Pretty/Not<br>□ Punctual Very/Pretty/Not<br><br><u>Bus is:</u><br>□ Fast Very/Pretty/Not<br>□ Comfortable Very/Pretty/Not<br>□ Cheap Very/Pretty/Not<br>□ Safe, Secure Very/Pretty/Not<br>□ Convenient Very/Pretty/Not<br>□ Relaxing Very/Pretty/Not<br>□ Green Very/Pretty/Not<br>□ Punctual Very/Pretty/Not	Each item was rated, ranking from 1 (very) to 3 (Not).<br>A score was computed by calculating the average of the 16 items.<br>(Cronbach's α=0.87)	Binary variable categorized at the median value (threshold=0.71).

**Relatives and friends' use of active transportation modes**			
Family's use of walking/cycling for transportation *(high vs. low)*	In your family, most of the adults use bike to reach their activities locations (work, study, shopping, visits, cinema, concert, bar, restaurant, ...):<br>□ Strongly agree<br>□ Agree<br>□ Neither agree nor disagree<br>□ Disagree<br>□ Strongly disagree<br><br>In your family, most of the adults walk to reach their activities locations (work, study, shopping, visits, cinema, concert, bar, restaurant, …):<br>□ Strongly agree<br>□ Agree<br>□ Neither agree nor disagree<br>□ Disagree<br>□ Strongly disagree	Mean of the two item questions ranking from 1 (Strongly agree) to 5 (strongly disagree). (Cronbach's α=0.58)	Binary variable categorized at the median value (threshold=0.3).
Friends' use of walking/cycling for transportation *(high vs. low)*	Most of your friends use bike to reach their activities locations (work, study, shopping, visits, cinema, concert, bar, restaurant, …):<br>□ Strongly agree<br>□ Agree<br>□ Neither agree nor disagree<br>□ Disagree<br>□ Strongly disagree<br><br>Your friends walk to reach their activities locations (work, study, shopping, visits, cinema, concert, bar, restaurant, …):<br>□ Strongly agree<br>□ Agree<br>□ Neither agree nor disagree<br>□ Disagree<br>□ Strongly disagree	Mean of the two item questions ranking from 1 (Strongly agree) to 5 (strongly disagree). (Cronbach's α=0.76)	Binary variable categorized at the median value (threshold=0.3).

Representation of physical activity			
Self-representation of an active person	Do you consider yourself as a physically active person:<br>□ Yes<br>□ No	-	Binary variable
Family representation of physical activity/sports	Did you grow up in a family how valued physical activity and more particularly sport and exercise<br>□ Yes, a lot<br>□ Yes a little bit<br>□ No	-	Binary variables categorized as high (Yes, a lot) versus low (else)

<u>Supplementary Table 2:</u> Unadjusted associations between perceived environmental, representation of transportation modes, physical activity and clusters of physical activity and sedentary behaviors. Results from multinomial logistic regression model using the cluster 5- active transportation as a reference. (N=23432)

	**Cluster 1**		**Cluster 2**		**Cluster 3**		**Cluster 4**		**Cluster 6**	
	Active occupation, low leisure time		*Sedentary occupation, low leisure time*		*Sedentary transportation*		*Sedentary occupation, high sedentary leisure*		*Active leisure*	
	OR	(CI 99%)	OR	(CI 99%)	OR	(CI 99%)	OR	(CI 99%)	OR	(CI 99%)
**Perception of the residential environment**										
Availability of destination *(high vs low)*	**0.46**	**(0.39 ; 0.54)**	**0.62**	**(0.53 ; 0.73)**	**0.43**	**(0.34 ; 0.54)**	0.82	(0.68 ; 1.01)	**0.60**	**(0.45 ; 0.79)**
Ease to walk on sidewalks *(high vs low)*	**0.52**	**(0.43 ; 0.63)**	**0.70**	**(0.58 ; 0.85)**	**0.47**	**(0.36 ; 0.61)**	0.86	(0.68 ; 1.08)	**0.45**	**(0.34 ; 0.62)**
Presence of bicycle paths *(high vs low)*	**0.45**	**(0.38 ; 0.53)**	**0.57**	**(0.49 ; 0.67)**	**0.44**	**(0.34 ; 0.57)**	**0.68**	**(0.56 ; 0.83)**	**0.49**	**(0.37 ; 0.66)**
Perceived pollution *(high vs low)*	**0.59**	**(0.50 ; 0.69)**	**0.69**	**(0.59 ; 0.81)**	**0.60**	**(0.46 ; 0.78)**	0.88	(0.73 ; 1.07)	**0.53**	**(0.39 ; 0.73)**
Perceived cleanliness of the neighborhood *(high vs low)*	**1.41**	**(1.18 ; 1.68)**	**1.39**	**(1.17 ; 1.64)**	1.28	(0.98 ; 1.68)	1.18	(0.95 ; 1.45)	**1.59**	**(1.14 ; 2.22)**
Presence of trees *(high vs low)*	0.92	(0.78 ; 1.09)	0.95	(0.81 ; 1.12)	1.00	(0.80 ; 1.43)	0.92	(0.75 ; 1.12)	1.07	(0.80 ; 1.43)
Presence of green spaces *(high vs low)*	0.91	(0.75 ; 1.11)	0.96	(0.79 ; 1.16)	0.89	(0.66 ; 1.18)	0.84	(0.67 ; 1.05)	1.12	(0.78 ; 1.59)
Aesthetics *(high vs low)*	**1.45**	**(1.22 ; 1.73)**	**1.40**	**(1.18 ; 1.65)**	**1.49**	**(1.13 ; 1.97)**	1.06	(0.86 ; 1.30)	**1.68**	**(1.20 ; 2.35)**
Traffic *(high vs low)*	1.27	(0.97 ; 1.67)	1.07	(0.97 ; 1.67)	**1.71**	**(1.18 ; 2.46)**	0.96	(0.69 ; 1.34)	1.22	(0.77 ; 1.91)
Criminality *(high vs low)*	**0.74**	**(0.63 ; 0.86)**	**0.83**	**(0.71 ; 0.96)**	**0.70**	**(0.56 ; 0.89)**	1.04	(0.86 ; 1.25)	**0.72**	**(0.55 ; 0.95)**
**Positive representations of transportation modes**										
Walking *(high vs low)*	**0.44**	**(0.37 ; 0.52)**	**0.48**	**(0.41 ; 0.57)**	**0.35**	**(0.26 ; 0.48)**	**0.50**	**(0.40 ; 0.62)**	0.79	(0.58 ; 1.07)
BCycling *(high vs low)*	**0.66**	**(0.56 ; 0.77)**	**0.61**	**(0.52 ; 0.72)**	**0.53**	**(0.42 ; 0.68)**	**0.54**	**(0.44 ; 0.65)**	0.80	(0.60 ; 1.06)
Individual motorized transportation modes *(high vs low)*	**1.41**	**(1.20 ; 1.66)**	**1.21**	**(1.04 ; 1.42)**	1.19	(0.93 ; 1.52)	**1.29**	**(1.07 ; 1.57)**	**1.59**	**(1.21 ; 2.09)**
Public transport *(high vs low)*	**0.74**	**(0.63 ; 0.86)**	0.90	(0.77 ; 1.05)	**0.58**	**(0.46 ; 0.73)**	0.89	(0.74 ; 1.08)	**0.75**	**(0.57 ; 0.99)**
**Relatives and friends' use of active transportation modes**										
Family's use of walking/cycling for transportation *(high vs low)*	**0.35**	**(0.29 ; 0.41)**	**0.41**	**(0.35 ; 0.49)**	**0.30**	**(0.24 ; 0.39)**	**0.45**	**(0.37 ; 0.55)**	**0.36**	**(0.27 ; 0.47)**
Friends' use of walking/cycling for	**0.43**	**(0.37; 0.51)**	**0.56**	**(0.47 ; 0.66)**	**0.37**	**(0.29 ; 0.47)**	**0.61**	**(0.50 ; 0.74)**	**0.43**	**(0.33 ; 0.58)**

transportation *(high vs low)*										
**Representation of physical activity**										
Self-representation of an active person (yes vs. no)	**0.64**	**(0.54 ; 0.76)**	**0.44**	**(0.37 ; 0.52)**	**0.53**	**(0.42 ; 0.68)**	**0.28**	**(0.23 ; 0.34)**	1.18	(0.86 ; 1.60)
Family representation of physical activity/sports (high vs. low)	**0.80**	**(0.64 ; 0.99)**	0.85	(0.69 ; 1.04)	0.93	(0.67 ; 1.28)	0.84	(0.65 ; 1.08)	1.30	(0.92 ; 1.84)

CI: confidence interval, OR: odds ratio
